# Supplementary material for: The rhg1‐a (Rhg1 low‐copy) nematode resistance source harbors a copia‐family retrotransposon within the Rhg1‐encoded α‐SNAP gene
Source: Plant Direct. 2019 Aug 28;3(8):e00164. doi: 10.1002/pld3.164 (PMC6712407; doi:10.1002/pld3.164)
Supplement: Supplementary file 3 [file PLD3-3-e00164-s003.pdf]

Response Letter:

8-July-2019

Dr. Macintosh -
Thank you for your handling of our manuscript Plant Direct: 2019-00299 entitled " The *rhg1-a* (*Rhg1* low-copy) nematode resistance source harbors a copia-family retrotransposon within the *Rhg1*-encoded α-SNAP gene". We are pleased to have made all recommended changes, and we now submit the attached revised manuscript for consideration. As part of our response we now, as suggested, include new data regarding a control, showing absence of the copia insert in *rhg1-b* (high copy number *Rhg1*). Each of our manuscript changes is noted in this response letter (below) and can readily identified in the attached document with tracked changes.

For the following text we pasted in, verbatim, all of your letter. For ease of reading we use a double-asterisk at the start of each of our intervening response paragraphs.

We hope you will agree that the manuscript is now in excellent shape for publication.
Thank you again for your work Plant Direct - it is much appreciated,
Andrew

From: "plantdirect@msubmit.net" <plantdirect@msubmit.net>
Reply-To: "plantdirect@wiley.com" <plantdirect@wiley.com>
Date: Wednesday, June 26, 2019 at 10:49 AM
To: Andrew Bent <afbent@wisc.edu>
Cc: "plantdirect@wiley.com" <plantdirect@wiley.com>
Subject: Plant Direct: 2019-00299: Decision Letter

June 25, 2019

Andrew F. Bent
University of Wisconsin - Madison
Department of Plant Pathology, 886 Russell Laboratories, 1630 Linden Drive
Madison, Wisconsin 53706-1598

RE: The rhg1-a (Rhg1 low-copy) nematode resistance source harbors a copia-family retrotransposon within the Rhg1-encoded α-SNAP gene

Dear Dr. Bent:

Thank you for submitting to Plant Direct. All required reviews have been returned and we have now finished our evaluation of your manuscript. In light of the reviewers' and editor's comments, further revisions are needed before the paper can be accepted for publication in Plant Direct.

Please view the editors' and reviewers' comments below and use their suggestions as a guide while you work on your revision.

When uploading the revised version of this article, please be sure to include the following:

-A word document that contains your response to the reviewers. You should respond to each reviewer comment and note the changes made to the manuscript. If you do not agree with a reviewer's comment and choose not to make a suggested revision, please explain why. Please try to provide as complete an answer as possible to each reviewer's criticisms.
-A tracked changes document with each change highlighted
- A clean version of the latest version of the manuscript

The reviewers were very positive, and the revisions requested are minor. I look forward to seeing the revised manuscript.

To upload your revision, please click the link below.
https://plantdirect.msubmit.net/cgi-bin/main.plex?el=A1Lr5HL3A6Zm1I5A9ftdy3O5TJDEhxUVi9NgqYyt7gZ

In order to provide as timely a service as possible, we ask that your revision is resubmitted within three months after receipt of this request. If an extension is needed, please send a request, along with a brief explanation, to the editorial office at plantdirect@wiley.com .

Please note that, in addition to publishing reviewer comments, the author's responses to review comments will also be published alongside the final version of the paper. If you would not like the author's responses to be published, please contact the editorial office at plantdirect@wiley.com .

Thank you very much for giving us an opportunity to review your work. I look forward to receiving the next version.

Sincerely,

Gustavo MacIntosh

Ivan Baxter

Editor, Plant Direct

-- Reviewer comments:
Reviewer #1:

In this manuscript, Bayless et al, report on the presence of a copia retrotransposon element within

the rhg1 gene, solely in the soybean accessions containing the rhg1-a haplotype. The authors performed various assays investigating potential associations between the presence of this TE and rhg1-a expression, and hence resistance to SCN. However, no such association can be established. While these results did not advance our understating of the functional role of rhg1 in SCN resistance, I believe this manuscript provides additional new insights into the unique structure characteristics of rhg1 locus in soybean, and hence merit publication.

**Thank you for your positive review of the manuscript.

It may be important for the authors to consider the potential association between DNA methylation, elongation rate of Pol II, and alternative splicing of rhg1-a in more thorough analysis in future analysis. DNA methylation may influence the recruitment of splicing factors to the pre-mRNA, impacting the elongation rate of Pol II, and hence exon inclusion/skipping on the rhg1-a mature mRNA.

**Yes - we now have a large study underway regarding DNA methylation at the *Rhg1* locus, and this is an excellent hypothesis for us to consider in that work.

Reviewer #2:

Can a positive control be shown for Fig.S1 using the same primers on WT or Rhg1-b genomic DNA? At minimum a there should be markings on the gel to illustrate the expected size and markings somewhere in figure 1 to denote the position of the primers.

**Yes, this is a helpful suggestion. We did a new experiment and added it to Fig. S1 (new Fig. S1 panel B). We also added a diagram to that figure showing the location of the PCR primers used in Fig. S1B and S1C.

Line 588-598 it is unclear whether the R, MR, MS, S has been developed by the authors, the previously studies or the USDA GRIN database. Is the classification of R, MR, MS and S consistent enough across studies to group the studies and collectively reclassify the results?

**The different research groups all determined female index for plants approximately 30 days after inoculation, which is a very commonly used SCN resistance metric. In those tests, results are normalized to the average number of mature/fertilized females that formed on the susceptible control within the experiment. So yes, although one cannot fully eliminate lab-to-lab and experiment-to-experiment variation, we do think it is valid to group and collectively reclassify. Of course there will always be a few false-positives and false-negatives in a dataset of over 500 soybean lines. We added a sentence to this effect at Lines 594-596.

A point of curiosity, can the authors share any insight as to why some "R" RAC- lines are resistant? Is this Rhg4 resistance? (Figure 4).

**We wondered the same thing. It probably is not *Rhg4* resistance (in the absence of *rhg1-a*), because multiple publications have reported that *Rhg4* gives at best "MR" when not coupled

with *rhg1-a*. We added the following sentences to the Discussion at lines 850-854: "The 5 lines from the GRIN database that had the SoySNP50K *rhg1-a* SNP signature and scored as "R" (SCN-resistant) despite lacking RAC merit future investigation. They may be a combination of plants mis-scored as "R", plants that unexpectedly carry *rhg1-b* or *rhg1-b*-derived variants despite their SNP signature, plants with novel *rhg1* alleles, and/or plants that carry combinations of other SCN resistance QTLs including possible novel QTLs."

-- Editor comments:
The reviewers were very positive, and the revisions requested are minor. I look forward to seeing the revised manuscript.

**Thank you.
